# Secondary cryofibrinogenemia is related to more severe microangiopathic involvement in systemic sclerosis: results from a retrospective observational study

**DOI:** 10.1007/s10067-025-07324-z

**Published:** 2025-01-20

**Authors:** Gilda Sandri, Gabriele Amati, Amelia Spinella, Patrizia Natali, Daria Debbia, Martina Orlandi, Ottavio Secchi, Benedetta Bongiovanni, Marco de Pinto, Maria Teresa Mascia, Dilia Giuggioli

**Affiliations:** 1 Rheumatology Unit, Scleroderma Unit, University Hospital of Modena, Via del Pozzo, 71–41125 Modena, Italy; 2 Department of Maternal, Child and Adult Medical and Surgical Sciences, University of Modena and Reggio Emilia, Modena, Italy; 3 Department of Laboratory Medicine and Pathological Anatomy, Azienda Ospedaliero- Universitaria E Azienda USL Di Modena, Ospedale Civile Di Baggiovara, Modena, Italy; 4 Rheumatology Unit, University of Modena and Reggio Emilia, Medical School, University Hospital of Modena, Policlinico of Modena, Modena, Italy

**Keywords:** Cryofibrinogen, Cryoproteins, Systemic sclerosis, Digital ulcers, Pulmonary arterial hypertension, Endothelin receptor antagonists

## Abstract

The aims of this study were to investigate the prevalence of cryofibrinogenemia in a cohort of patients with systemic sclerosis (SSc) regardless of clinical manifestations, who were admitted to our hospital and determine the associations among CF positivity, disease features and ongoing therapies. This was a monocentric and retrospective study. The inclusion criteria were a diagnosis of SSc (according to the ACR/EULAR 2013 classification criteria), regular administration of i.v. prostanoids, and CF testing between February 2020 and February 2022. Data on demographic, clinical, and immunological features and ongoing treatments were retrospectively collected. Categorical data were compared with the chi-square test or Fisher’s exact test, while quantitative variables comparisons were carried out with Student’s t test or Mann‒Whitney test. In total, 101 SSc patients were ultimately enrolled. The majority of patients were female (92.1%) and had the limited cutaneous form of SSc (81.2%). CF positivity was observed in 69.3% of the patients, whereas only 9% presented cryoglobulins and CF. CF positivity was negatively associated to RNAP3 antibodies (*p* = 0.027). No direct associations with specific clinical phenotypes were observed. No associations with immunosuppressive treatments were identified, however a positive association with nifedipine administration (*p* = 0.040), and a negative association with endothelin receptor antagonists (ERAs) plus phosphodiesterase-5 (PDE5) inhibitors regimen (*p* = 0.031) were observed. Macitentan administration was also associated to CF cryocrit ≥ 1% (*p* = 0.045). Among patients who were not treated with ERAs, an estimated pulmonary artery systolic pressure ≥ 30 mmHg was significantly associated with CF positivity (*p* = 0.025). Moreover, a cryocrit ≥3% was associated with a relative risk of 3.44 (95% CI 1.26–9.39, *p* = 0.016) for digital amputation and 5.17 (95% CI 1.18–22.6, *p* = 0.029) for death. Isolated CF is a frequent phenomenon observed in SSc patients and is associated with a higher administration of vasoactive drugs, probably identifying a SSc clinical phenotype with a more severe microvascular involvement. Moreover, a higher cryocrit is associated with an increased risk of death and digital amputations. Screening SSc patients for CF would represent an opportunity to provide better therapeutic approaches by anticipating ERA administration in an earlier phase, thereby preventing the manifestation of severe microvascular involvement.

**Table:** 

**Key Points** • *Cryofibrinogen is a cryoprotein that can cause microangiopathic damage.* • *Isolated cryofibrinogenemia is common in patients with systemic sclerosis.* • *SSc patients should be tested for cryofibrinogen because a high cryocrit (≥ 3%) is associated with death and/or digital amputation due to necrosis.* • *Cryofibrinogen is associated with indirect markers of pulmonary arterial hypertension in patients not treated with endothelin receptor agonists (ERAs).* • *ERAs could play a role in preventing cryofibrinogen damage.*

**Supplementary Information:**

The online version contains supplementary material available at 10.1007/s10067-025-07324-z.

## Introduction

Cryofibrinogenemia refers to a condition characterized by the presence of blood cryofibrinogen (CF), a cryoprotein that precipitates in the plasma when cooled and dissolves again when it is rewarmed. Distinguish CF from cryoglobulins (CGs) is fundamental because CGs are detectable in both plasma and serum, and are frequently associated with the presence of CF, potentially leading to misdiagnosis of the related cryoproteinemic syndromes [1]. Despite being found in almost 3% of the healthy population, CF seems to increase the risk of thrombosis [1–7]. The pathogenesis of cryofibrinogenemia may be related to a reduction in the fibrinolytic process or an increase in thrombin-binding capacity; both hypotheses lead to increased viscosity, vascular stasis, and reflex vasospasm [1–5].

The clinical manifestations of cyrofibrinogenemia include purpura, Raynaud’s phenomenon (RP), livedo reticularis, cold intolerance, skin necrosis or ulcerations, and gangrene [8]. This condition has also been associated with other disorders, such as autoimmune diseases; in this case the condition is classified as “secondary” [2, 8–10].

In our previous study, we found a high prevalence of SSc in a sample of 103 patients admitted to our hospital in whom CF was determined; in particular 19 out of 27 patients affected by SSc, were found to be positive to CF and the 77% of those presented CF without the association with CGs [11].

Systemic sclerosis (SSc) is a rare connective tissue disease characterized by early and severe microangiopathic damage, which leads to fibrosis of the skin and internal organs via inflammatory and autoimmune processes [12–15]. The more severe complications of microcirculatory involvement in SSc patients include digital ulcers (DUs), pulmonary arterial hypertension (PAH), and scleroderma renal crisis (SRC), which are associated with platelet activation, aggregation, and thrombotic events. Those complications are primarily treated with vasoactive drugs [16, 17].

Certain clinical features, particularly vascular complications, are shared by both CF and SSc. Moreover, the role of thrombosis on pathophysiology of SSc have been focused on a recent review [18].

In literature, only few reports describe the association of CF with SSc. In a recent retrospective study, the prevalence of CF was reported to be 50% among 75 SSc patients, that is significantly greater than the one reported in the general population. However, this phenomenon did not seem to affect survival in this population [19].

The aim of the present study was to investigate the prevalence of CF in a cohort of SSc patients admitted to our hospital regardless of their clinical manifestations and the associations between CF positivity and all other features, with a focus on clinical manifestations and ongoing therapies, including vasoactive drugs which have never been taken into account in previous studies.

The primary endpoint was the prevalence of CF positivity in a cohort of SSc patients and the associations between the presence of CF and specific SSc manifestations, comorbidities and ongoing therapies.

The secondary endpoint was to evaluate the risk of death and amputations according to cryocrit titer.

## Methods

### Population and study design

This was a monocentric, retrospective study conducted in SSc patients followed by the Scleroderma Unit of University Hospital of Modena, a tertiary referral center for SSc.

The inclusion criteria were a diagnosis of SSc according to the current American College of Rheumatology (ACR)/European Alliance of Associations for Rheumatology (EULAR) 2013 classification criteria [20], regular administration of i.v. prostanoids (one infusion every six weeks), and CF testing between February 2020 to and February 2022. The exclusion criteria were the presence of an indeterminate CF test result or over 75% of missing data on any of the variables of interest in this study.

The study was approved by the local Ethics Committee (Comitato Etico Area Vasta Emilia Nord, protocol number 275/16), and written informed consent was obtained from all participants.

### Data collection

Data collection was managed with an electronic case report form. Data on the demographic and clinical variables of interest in this study were obtained from SSc patients and included age at the time of study inclusion (CF sampling), sex, age at diagnosis, disease duration (years), cutaneous form of the disease (diffuse (dsSSc) or limited cutaneous SSc (lcSSc)), estimated pulmonary artery systolic pressure (ePASP, mmHg)—assessed via transthoracic echocardiography within a 1-year window –, established diagnosis of pulmonary arterial hypertension (PAH) assessed via right heart catheterization, presence of interstitial lung disease (ILD) as diagnosed on high-resolution computed tomography (HRCT), history of DUs, amputations (Amp), scleroderma renal crisis (SRC), calcinosis, arthritis, esophagopathy, telangiectasias, history of inflammatory bowel disease, history of neoplasms, and presence of a clear overlapping syndrome. Regarding the laboratory findings, CGs (typing and cryocrit), CF (cryocrit), and rheumatoid factor (RF, titer) evaluations were performed with the same blood sample. Antinuclear antibody (ANA) and extractable nuclear antigen (ENA) patterns, anti-citrullinated peptide antibodies (ACPAs), and anti-phospholipid antibodies were collected. The patients were also tested for the presence of hepatitis C virus (HCV) and/or hepatitis B virus (HBV) infection. SSc therapies administered prior to CF and CG blood sampling were considered ongoing therapies.

### Cryoprecipitate detection and level

CF and CG tests were performed according to Natali et al. [11]. Briefly, all blood collection materials (needles, syringes, tubes, etc.) were first prewarmed at 37 °C. The patients’ blood samples were collected in 10 mL tubes (BD Vacutainer tube, BD Company, Plymouth, UK); this volume excluded that of the anticoagulant and separator gel for CG but did include the volume of EDTA as an anticoagulant for CF.

The next steps were similar for detecting the presence of both CF and CG. After the test tubes were placed in a specific heated device suitable for maintaining a temperature of 37 °C, they were promptly dispatched to the laboratory, where they were kept in an incubator at 37 °C. The sera required a minimum of 1 to 2 h of incubation for clotting and to avoid CG precipitation. The plasma and serum were centrifuged for 15 min at 1,500 × g in a thermostatic centrifuge, and the supernatant was separated into two tubes and then kept at 4 °C for 7 days. The presence of cryoprecipitates in the serum and plasma was evident through manual observation: CF precipitates only in plasma, whereas CGs precipitate in both serum and plasma. Once a cryoprecipitate formed, the test tube was warmed back to 37 °C to verify dissolution. Samples containing particulate or lipemic matter, either hemolyzed or strongly icteric, were discarded. The cryoprecipitates were then isolated via a refrigerated centrifuge (15 min, 1,500 × g, 4 °C).

The precipitates were purified via 3 washes with cold phosphate-buffered saline (PBS) or saline solution at 4 °C. After each wash, the samples were centrifuged again at 1,500 × g for 15 min at 4 °C. Following the last wash, 500 μL of preheated physiological solution was added to the pellet, after which the tubes were incubated at 37 °C for at least 2 h, through which the precipitate could dissolve for further analysis. CF and CGs were then identified via immunofixation (IFE) (Hydragel IF4, Sebia, Lisses, FR) with specific antisera: antifibrinogen antisera (Dako-Agilent, CA, USA) for characterizing CF and anti-IgG, anti-IgA, anti-IgM, anti-κ, and anti-λ antisera (Sebia, Lisses, FR) for characterizing CGs according to the Brouet classification [21].

The percentages of both the CF and CG cryoprecipitates relative to the plasma or serum volume were measured following 15 min of centrifugation at 500 × g in a graduated Wintrobe tube measuring 6.5 × 100 mm (Laboindustria SpA, Padova, Italy). The cryocrit was assessed up to a sensitivity of 1%; lower values are reported as < 1%.

### Statistical analysis

Categorical variables are described as frequencies, whereas quantitative variables are reported as the means and standard deviations of the means. The Shapiro–Wilk test was conducted to determine the normality of the distribution of the quantitative data. For data conforming to a normal distribution, comparisons between groups were carried out with Student’s t test; otherwise, the Mann‒Whitney test was performed. Categorical data were compared with the chi-square test or Fisher’s exact test. Statistical significance was defined as a p value lower than or equal to 0.05. The relative risk (RR), its standard error and 95% confidence interval were calculated via Altman analysis [22]. Statistical analyses were performed with JASP Team (2022) (Version 0.16.3, Netherlands) statistical software.

For the evaluation of the primary outcome, the data were initially analyzed regarding CF positivity (CF + vs CF-) and then via a subset analysis based on the cryocrit (CF < 1% vs. CF ≥ 1%). Furthers subset analysis were carried out according to the significant associations. For the evaluation of the secondary outcome, the frequencies of digital amputation and death were described according to cryocrit titer and then the relative risk of them was calculated according to a cryocrit titer corresponding to at least 50% of prevalence of one of the outcomes of interest (digital amputation or death).

## Results

### Descriptive statistics of the sample

A total of 103 patients were screened, but 2 patients were excluded because of indeterminate CF test result.

One hundred one patients were ultimately enrolled in this study; the majority were female (92.1%) and affected by a limited cutaneous subset of the disease (81.2%). A total of 69.3% of patients were positive for CF; the CF cryocrit was < 1% and ≥ 1% in respectively 62.3% and 37.7% of patients. Furthermore, only 9% of patients presented with both CF and CGs (Table 1). In only one patient was impossible to trace the CF cryocrit, so it was excluded from the further evaluation requiring CF cryocrit.

**Table 1:** Demographic and clinical data of the included population

	Missing	General (*n* = 101)
Mean age (years ± SD)	0	61.73 ± 14.42
Mean age at diagnosis (years ± SD)	0	51.47 ± 15.54
Mean disease duration (years ± SD)	0	10.71 ± 9.10
Female sex (*n*, %)	0	93 (92.1%)
Diffuse cutaneous SSc (*n*, %)	0	20 (19.8%)
CF positive (*n*, %)	0	70 (69.3%)
Diffuse cutaneous SSc (*n*, %)	1	26 (37.7%)
CG positive (*n*, %)	1	9 (9.0%)
CG cryocrit ≥ 1% (*n*, %)	0	2 (22.2%)
RF positive (cut off 15 IU/mL) (*n*, %)	1	23 (23.0%)
Scl-70 positive (*n*, %)	0	40 (39.6%)
ACA positive (*n*, %)	0	39 (38.6%)
Anti-RNAP3 positive (*n*, %)	0	3 (3.0%)
Anti-fibrillarin positive (*n*, %)	0	3 (3.0%)
Anti-U1RNP positive (*n*, %)	0	3 (3.0%)
Anti-Th/To positive (*n*, %)	0	3 (3.0%)
Anti-Ku positive (*n*, %)	0	2 (2.0%)
Anti-MDA5 positive (*n*, %)	0	1 (1.0%)
Anti-PmScl positive (*n*, %)	0	1 (1.0%)
AMA positive (*n*, %)	0	6 (6.0%)
Anti-Ro/SSA positive (*n*, %)	0	17 (16.8%)
Anti-La/SSB positive (*n*, %)	0	5 (5.0%)
ACPA positive (*n*, %)	9	3 (3.3%)
aPL antibody positive (*n*, %)	0	13 (18.6%)
ACLA IgM positive (*n*, %)	0	12 (11.9%)
ACLA IgG positive (*n*, %)	0	2 (2.0%)
B2GP1 IgM positive (*n*, %)	0	4 (4.0%)
B2GP1 IgG positive (*n*, %)	0	1 (1.0%)
LAC positive (*n*, %)	11	2 (2.2%)
HCV positive (*n*, %)	37	2 (3.1%)
HBV positive (*n*, %)	35	6 (9.1%)
Presence of ILD (*n*, %)	0	63 (62.4%)
Presence of ePASP > 30 mmHg (*n*, %)	0	44 (43.6%)
Presence of PAH (*n*, %)	89	7 (58.3%)
Raynaud’s phenomenon (*n*, %)	0	101 (100.0%)
DUs (present/past) (*n*, %)	0	55 (54.5%)
History of amputations (*n*, %)	0	16 (15.8%)
History of scleroderma renal crisis (*n*, %)	0	2 (2.0%)
History of calcinosis (*n*, %)	0	30 (29.7%)
History of arthritis (*n*, %)	0	12 (11.9%)
Esophagopathy (*n*, %)	0	65 (64.4%)
Telangectasias (*n*, %)	0	55 (54.5%)
History of neoplasm (*n*, %)	0	20 (19.8%)
Presence of overlap syndrome (*n*, %)	0	35 (34.7%)
Death (*n*, %)	0	6 (6.0%)
Prostanoids (*n*, %)	0	89 (90.8%)
Iloprost (*n*, %)	0	86 (87.8%)
Alprostadil (*n*, %)	0	3 (3.1%)
Calcium channel blockers (*n*, %)	0	76 (77.6%)
Nifedipine (*n*, %)	0	40 (40.8%)
Amlodipine (*n*, %)	0	28 (28.6%)
Diltiazem (*n*, %)	0	4 (4.1%)
Felodipine (*n*, %)	0	3 (3.1%)
Antiplatelets (*n*, %)	0	44 (44.9%)
Low-dose acetylsalicylate (*n*, %)	0	41 (41.8%)
Ticagrelor (*n*, %)	0	1 (1.0%)
Clopidogrel (*n*, %)	0	2 (2.0%)
Anticoagulant (*n*, %)	0	6 (6.1%)
Dabigatran (*n*, %)	0	2 (2.0%)
Apixaban (*n*, %)	0	3 (3.1%)
Edoxaban (*n*, %)	0	1 (1.0%)
ERAs (*n*, %)	0	44 (44.9%)
Sildenafil (*n*, %)	0	9 (9.2%)
Sildenafil + ERAs (*n*, %)	0	7 (7.1%)
Immunosuppressants (*n*, %)	0	39 (39.8%)
MMF (*n*, %)	0	24 (24.5%)
HCQ (*n*, %)	0	13 (13.3%)
MTX (*n*, %)	0	3 (3.1%)
AZA (*n*, %)	0	1 (1.0%)
LEF (*n*, %)	0	1 (1.0%)
RTX (*n*, %)	0	8 (8.2%)
Low-dose steroids (*n*, %)	0	42 (42.9%)
Nintedanib (*n*, %)	0	3 (3.1%)

*ACA* anti-centromere antibody, *ACLA* anti-cardiolipin antibody, *ACPA* anti-citrullinated peptide antibody, *AZA* azathioprine, *B2GP1* anti-beta2glycoprotein 1 antibody, *CCBs* calcium channel blockers, *CF* cryofibrinogen/cryofibrinogenemia, *CG* cryoglobulins/cryoglobulinemia, *dsSSc* diffuse cutaneous subset, *DUs* digital ulcers, *ePASP* estimated pulmonary arterial pressure, *ERAs* endothelin receptor antagonists, *HBV* hepatitis B virus, *HCQ* hydroxychloroquine, *HCV* hepatitis C virus, *ILD* interstitial lung disease, *LAC* lupus anticoagulant, *LEF* leflunomide, *MMF* mycophenolate mofetile, *MTX* metothrexate, *PAH* pulmonary arterial hypertension, *PDE5i* phosphodiesterase 5 inhibitor, *RF* rheumatoid factor, *RNAP3* anti-RNA polymerase 3 antibody, *RTX* rituximab, anti-CD20 antibody, *Scl70* anti-topoisomerase I antibody, *SRC* scleroderma renal crisis

SSc-specific autoantibodies positivity was balanced between anti-topoisomerase I antibodies (Scl70) and anti-centromere antibodies (ACA), by each representing almost the 39% of the overall positivity. Only 3% of patients were positive for RNA polymerase 3 antibodies (RNAP3) and the remaining patients were positive for other autoantibodies (Table 1).

The disease involvement was comparable with the principal cohorts in literature, with a prevalence of ∼50% of digital ulcers and a slightly higher prevalence of interstitial lung disease (∼70%). Seven out of 12 patients who performed right heart catheterization resulted to have pulmonary arterial hypertension, but by considering ePASP as an indirect marker of PAH, 43% of patients resulted to have ePASP ≥ 30 mmHg (Table 1).

In the whole sample, 6 patients (5.9%) died, and 16 patients (15.8%) required digital amputation due to complications of digital ulcers (Table 1).

### Comparison of demographic and clinical characteristics between CF-positive and CF-negative patients:

No associations were identified between CF positivity and Scl70 positivity (CF + Scl70 + 41.4% vs CF-Scl70 + 35.5%), *p* = 0.338), and ACA positivity (CF + ACA + 38.6% vs CF-ACA + 38.7%, *p* = 0.989), but a negative association was found with RNAP3 positivity (CF + RNAP3 + 0.0% vs CF-RNAP3 + 9.7%, *p* = 0.027).

No associations emerged by the comparison between CF positivity and presence of ILD (CF + ILD + 62.9% vs CF-ILD + 61.3%, *p* = 0.881), history of DUs (CF + DUs + 54.2% vs CF-DUs + 54.8%, *p* = 0.826), PAH (CF + PAH + 55.6% vs CF-PAH + 66.7%, *p* = 1.00), ePASP ≥ 30 mmHg (CF + ePASP + 44.3% vs CF-ePASP + 42.9%, *p* = 0.826), history of digital amputations (CF + Amp + 17.1% vs CF-Amp + 12.9%), and death (CF + Death + 8.6% vs CF-Death + 3.2%).

No associations were identified between CF positivity and all other clinical features, autoantibodies, or immunosuppressive therapy regimens reported among the study patients (Table 2).

**Table 2:** Comparison of demographic and clinical characteristics between CF-positive and CF-negative patients

	Missing	CF-positive (*n* = 70)	CF-negative (*n* = 31)	*p*
Mean age (years ± SD)	0	61.73 ± 14.42	60.29 ± 12.44	0.369
Mean age at diagnosis (years ± SD)	0	51.47 ± 15.54	48.29 ± 11.82	0.247
Mean disease duration (years ± SD)	0	10.71 ± 9.10	12.29 ± 8.61	0.308
Female sex (*n*, %)	0	63 (90.0%)	30 (96.8%)	0.429
Diffuse cutaneous SSc (*n*, %)	0	14 (20.0%)	6 (19.4%)	0.940
CF cryocrit ≥ 1% (*n*, %)	1	26 (37.1%)	-	-
CG positive (*n*, %)	1	9 (86.6%)	0 (0.0%)	*0.054*
CG cryocrit ≥ 1% (*n*, %)	0	2 (25.0%)	0 (0.0%)	1.00
RF positive (cut off 15 IU/mL) (*n*, %)	1	17 (24.6%)	6 (19.4%)	0.562
Scl-70 positive (*n*, %)	0	29 (41.4%)	11 (35.5%)	0.667
ACA positive (*n*, %)	0	27 (38.6%)	12 (38.7%)	0.989
Anti-RNAP3 positive (*n*, %)	0	0 (0.0%)	3 (9.7%)	**0.027**
Anti-fibrillarin positive (*n*, %)	0	3 (4.3%)	0 (0.0%)	0.551
Anti-U1RNP positive (*n*, %)	0	3 (4.3%)	0 (0.0%)	0.551
Anti-Th/To positive (*n*, %)	0	2 (2.9%)	1 (3.2%)	1.00
Anti-Ku positive (*n*, %)	0	2 (2.9%)	0 (0.0%)	1.00
Anti-MDA5 positive (*n*, %)	0	1 (1.4%)	0 (0.0%)	1.00
Anti-PmScl positive (*n*, %)	0	1 (1.4%)	0 (0.0%)	1.00
AMA positive (*n*, %)	0	4 (5.7%)	2 (6.5%)	1.00
Anti-Ro/SSA positive (*n*, %)	0	10 (14.3%)	7 (22.6%)	0.304
Anti-La/SSB positive (*n*, %)	0	2 (2.9%)	3 (9.7%)	0.167
ACPA positive (*n*, %)	9	2 (3.2%)	1 (3.3%)	1.00
aPL antibody positive (*n*, %)	0	13 (18.6%)	5 (16.1%)	0.767
ACLA IgM positive (*n*, %)	0	9 (12.9%)	3 (9.7%)	0.751
ACLA IgG positive (*n*, %)	0	1 (1.4%)	1 (3.2%)	0.522
B2GP1 IgM positive (*n*, %)	0	3 (4.3%)	1 (3.2%)	1.00
B2GP1 IgG positive (*n*, %)	0	1 (1.4%)	0 (0.0%)	1.00
LAC positive (*n*, %)	11	2 (3.2%)	0 (0.0%)	1.00
HCV positive (*n*, %)	37	2 (4.5%)	0 (0.0%)	1.00
HBV positive (*n*, %)	35	4 (8.9%)	2 (9.5%)	1.00
Presence of ILD (*n*, %)	0	44 (62.9%)	19 (61.3%)	0.881
Presence of ePASP > 30 mmHg (*n*, %)	0	31 (44.3%)	13 (42.9%)	0.826
Presence of PAH (*n*, %)	89	5 (55.6%)	2 (66.7%)	1.00
Raynaud’s phenomenon (*n*, %)	0	70 (100.0%)	31 (100.0%)	-
DUs (present/past) (*n*, %)	0	38 (54.2%)	17 (54.8%)	0.959
History of amputations (*n*, %)	0	12 (17.1%)	4 (12.9%)	0.770
History of scleroderma renal crisis (*n*, %)	0	1 (1.4%)	1 (3.2%)	0.522
History of calcinosis (*n*, %)	0	17 (24.3%)	13 (41.9%)	*0.073*
History of arthritis (*n*, %)	0	9 (12.9%)	3 (9.7%)	0.751
Esophagopathy (*n*, %)	0	45 (64.3%)	20 (64.5%)	0.982
Telangectasias (*n*, %)	0	38 (54.3%)	17 (54.8%)	1.00
History of neoplasm (*n*, %)	0	13 (18.5%)	7 (22.6%)	0.641
Presence of overlap syndrome (*n*, %)	0	22 (31.4%)	13 (41.9%)	0.306
Death (*n*, %)	0	6 (8.6%)	1 (3.2%)	0.443
Prostanoids (*n*, %)	0	61 (91.0%)	28 (90.3%)	1.00
Iloprost (*n*, %)	0	58 (86.7%)	28 (90.3%)	0.747
Alprostadil (*n*, %)	0	3 (4.5%)	0 (0.0%)	0.549
Calcium channel blockers (*n*, %)	0	54 (80.6%)	2 (71.0%)	0.288
Nifedipine (*n*, %)	0	32 (47.8%)	8 (25.8%)	**0.040**
Amlodipine (*n*, %)	0	16 (23.9%)	12 (38.7%)	0.131
Diltiazem (*n*, %)	0	4 (6.0%)	0 (0.0%)	0.304
Felodipine (*n*, %)	0	2 (3.2%)	1 (3.0%)	1.00
Antiplatelets (*n*, %)	0	34 (50.7%)	10 (32.3%)	*0.087*
Low-dose acetylsalicylate (*n*, %)	0	32 (47.8%)	9 (29.0%)	*0.080*
Ticagrelor (*n*, %)	0	1 (1.5%)	0 (0.0%)	1.00
Clopidogrel (*n*, %)	0	1 (1.5%)	1 (3.2%)	0.535
Anticoagulant (*n*, %)	0	4 (6.0%)	2 (6.5%)	1.00
Dabigatran (*n*, %)	0	1 (1.5%)	1 (3.2%)	0.535
Apixaban (*n*, %)	0	2 (3.0%)	1 (3.2%)	1.00
Edoxaban (*n*, %)	0	1 (1.5%)	0 (0.0%)	1.00
ERAs (*n*, %)	0	28 (41.8%)	16 (51.6%)	0.363
Sildenafil (*n*, %)	0	4 (6.0%)	5 (16.1%)	0.136
Sildenafil + ERAs (*n*, %)	0	2 (3.0%)	5 (16.1%)	***0.031***
Immunosuppressants (*n*, %)	0	27 (40.3%)	12 (38.7%)	0.881
MMF (*n*, %)	0	17 (25.4%)	7 (22.6%)	0.765
HCQ (*n*, %)	0	7 (10.5%)	6 (19.4%)	0.336
MTX (*n*, %)	0	1 (1.5%)	2 (6.5%)	0.234
AZA (*n*, %)	0	1 (1.5%)	0 (0.0%)	1.00
LEF (*n*, %)	0	1 (1.5%)	0 (0.0%)	1.00
RTX (*n*, %)	0	5 (7.5%)	3 (9.7%)	0.705
Low-dose steroids (*n*, %)	0	31 (46.3%)	11 (35.5%)	0.316
Nintedanib (*n*, %)	0	2 (3.0%)	1 (3.2%)	1.00

*ACA* anti-centromere antibody, *ACLA* anti-cardiolipin antibody, *ACPA* anti-citrullinated peptide antibody, *AZA* azathioprine, *B2GP1* anti-beta2glycoprotein 1 antibody, *CCBs* calcium channel blockers, *CF* cryofibrinogen/cryofibrinogenemia, *CG* cryoglobulins/cryoglobulinemia, *dsSSc* diffuse cutaneous subset, *DUs* digital ulcers, *ePASP* estimated pulmonary arterial pressure, *ERAs* endothelin receptor antagonists, *HBV* hepatitis B virus, *HCQ* hydroxychloroquine, *HCV* hepatitis C virus, *ILD* interstitial lung disease, *LAC* lupus anticoagulant, *LEF* leflunomide, *MMF* mycophenolate mofetile, *MTX* metothrexate, *PAH* pulmonary arterial hypertension, *PDE5i* phosphodiesterase 5 inhibitor, *RF* rheumatoid factor, *RNAP3* anti-RNA polymerase 3 antibody, *RP* Raynaud’s Phenomenon, *RTX* rituximab, anti-CD20 antibody, *Scl70* anti-topoisomerase I antibody, *SRC* scleroderma renal crisis

The presence of CF was associated with ongoing vasoactive treatment, particularly a positive association with nifedipine-based (CF + Nif + 47.8% vs. CF-Nif + 25.8%, *p* = 0.040) and a negative association with endothelin receptor antagonists (ERAs) plus phosphodiesterase 5 inhibitors (PDE5i) -based therapy (CF + ERA + PDE5i + 3.0% vs. CF-ERAs + PDE5i + 16.1%, *p* = 0.031) (Table 2).

### Comparison of demographic and clinical characteristics between patients with CF cryocrit ≥ 1% and < 1%

No associations were found between groups of patients with CF cryocrit higher than or equal to 1% and CF cryocrit lower than 1% about clinical features, autoantibodies and immunosuppressant therapies.

A cryocrit higher than or equal to 1% was associated to a higher frequency of Macitentan administration (CF ≥ 1% and Macitentan + 21.7% vs CF < 1% and Macitentan + 4.7%, *p* = 0.045) (Table 3).

**Table 3:** Comparisons between patients with a CF cryocrit ≥ 1% and those with a CF cryocrit < 1%

	Missing	CF cryocrit ≥ 1% (*n* = 26)	CF cryocrit < 1% (*n* = 43)	*p*
Mean age (years ± SD)	0	61.81 ± 15.61	61.26 ± 13.69	0.878
Mean age at diagnosis (years ± SD)	0	49.96 ± 18.24	52.00 ± 13.81	0.601
Mean disease duration (years ± SD)	0	12.47 ± 10.79	9.57 ± 7.93	0.204
Female sex (*n*, %)	0	24 (92.3%)	38 (88.4%)	0.703
Diffuse cutaneous SSc (n, %)	0	6 (37.5%)	7 (16.3%)	0.535
CG positive (%)	0	5 (19.2%)	4 (9.3%)	0.282
CG cryocrit ≥ 1% (n, %)	0	2 (40.0%)	0 (0.0%)	1.00
RF positive (cut off 15 IU/mL) (n, %)	1	5 (20.0%)	12 (27.9%)	0.468
Scl-70 positive (n, %)	0	14 (53.8%)	14 (32.6%)	*0.081*
ACA positive (n, %)	0	11 (42.3%)	16 (37.2%)	0.674
Anti-RNAP3 positive (n, %)	0	0 (0.0%)	0 (0.0%)	-
Anti-fibrillarin positive (n, %)	0	0 (0.00%)	3 (7.0%)	0.285
Anti-U1RNP positive (n, %)	0	1 (3.9%)	2 (4.7%)	1.00
Anti-Th/To positive (n, %)	0	0 (0.0%)	2 (4.7%)	0.523
Anti-Ku positive (n, %)	0	1 (3.9%)	1 (2.3%)	1.00
Anti-MDA5 positive (n, %)	0	0 (0.0%)	1 (2.3%)	1.00
Anti-PmScl positive (n, %)	0	0 (0.0%)	1 (2.3%)	1.00
AMA positive (n, %)	0	1 (3.9%)	3 (7.0%)	1.00
Anti-Ro/SSA positive (n, %)	0	3 (11.5%)	7 (16.3%)	0.732
Anti-La/SSB positive (n, %)	0	1 (3.9%)	1 (2.3%)	1.00
ACPA positive (n, %)	8	1 (4.8%)	1 (2.5%)	1.00
aPL antibody positive (n, %)	0	6 (23.1%)	6 (14.0%)	0.347
ACLA IgM positive (n, %)	0	4 (15.4%)	5 (11.6%)	0.720
ACLA IgG positive (n, %)	0	0 (0.0%)	1 (2.3%)	1.00
B2GP1 IgM positive (n, %)	0	1 (3.8%)	1 (2.3%)	1.00
B2GP1 IgG positive (n, %)	0	1 (3.8%)	0 (0.0%)	0.377
LAC positive (n, %)	7	1 (4.2%)	1 (2.6%)	1.00
HCV positive (n, %)	25	2 (11.8%)	0 (0.0%)	0.144
HBV positive (n, %)	24	3 (16.7%)	1 (3.7%)	0.286
Presence of ILD (n, %)	0	15 (57.7%)	28 (65.1%)	0.537
Presence of ePASP > 30 mmHg (n, %)	0	15 (57.7%)	15 (34.8%)	*0.064*
Presence of PAH (n, %)	61	1 (50.0%)	3 (50.0%)	1.00
Raynaud’s phenomenon (n, %)	0	26 (100.0%)	43 (100.0%)	-
DUs (present/past) (n, %)	0	16 (61.5%)	21 (48.8%)	0.305
History of amputations (n, %)	0	7 (26.9%)	5 (11.6%)	0.188
History of scleroderma renal crisis (n, %)	0	1 (3.8%)	0 (0.0%)	0.377
History of calcinosis (n, %)	0	5 (19.2%)	11 (25.6%)	0.545
History of arthritis (n, %)	0	5 (19.2%)	4 (9.3%)	0.282
Esophagopathy (n, %)	0	14 (53.8%)	30 (69.8%)	0.182
Telangectasias (n, %)	0	16 (61.5%)	22 (51.2%)	0.401
History of neoplasm (n, %)	0	5 (19.2%)	8 (18.6%)	1.00
Presence of overlap syndrome (n, %)	0	7 (26.9%)	15 (34.9%)	0.492
Death (n, %)	0	3 (11.5%)	3 (7.0%)	0.665
Prostanoids (n, %)	0	20 (87.0%)	40 (93.0%)	0.413
Iloprost (n, %)	0	20 (87.0%)	37 (86.0%)	1.00
Alprostadil (n, %)	0	0 (0.0%)	3 (7.0%)	0.546
Calcium channel blockers	0	19 (82.6%)	35 (81.4%)	1.00
Nifedipine (n, %)	0	9 (39.1%)	23 (53.5%)	0.266
Amlodipine (n, %)	0	8 (34.8%)	8 (18.6%)	0.144
Diltiazem (n, %)	0	1 (4.3%)	3 (7.0%)	1.00
Felodipine (n, %)	0	1 (4.3%)	1 (2.3%)	1.00
Antiplatelets (n, %)	0	15 (65.2%)	18 (41.9%)	*0.071*
Low-dose acetylsalicylate (n, %)	0	14 (60.9%)	17 (39.5%)	*0.098*
Ticagrelor (n, %)	0	1 (4.4%)	0 (0.0%)	0.348
Clopidogrel (n, %)	0	0 (0.0%)	1 (2.3%)	1.00
Anticoagulant (n, %)	0	2 (8.7%)	2 (4.7%)	0.606
Dabigatran (n, %)	0	1 (4.3%)	0 (0.0%)	0.348
Apixaban (n, %)	0	0 (0.0%)	2 (4.7%)	0.539
Edoxaban (n, %)	0	1 (4.3%)	0 (0.0%)	0.348
ERAs (n, %)	0	11 (47.8%)	16 (37.2%)	0.403
Bosentan (n, %)	0	6 (26.1%)	14 (32.6%)	0.586
Macitentan (n, %)	0	5 (21.7%)	2 (4.7%)	**0.045**
Sildenafil (n, %)	0	1 (4.3%)	3 (7.0%)	1.00
Sildenafil + ERAs (n, %)	0	1 (4.3%)	1 (2.3%)	1.00
Immunosuppressants (n, %)	0	9 (39.1%)	18 (41.9%)	0.830
MMF (n, %)	0	4 (17.4%)	13 (30.2%)	0.256
HCQ (n, %)	0	4 (17.4%)	3 (7.0%)	0.227
MTX (n, %)	0	0 (0.0%)	1 (2.3%)	1.00
AZA (n, %)	0	0 (0.0%)	1 (2.3%)	1.00
LEF (n, %)	0	1 (4.3%)	0 (0.0%)	0.348
RTX (n, %)	0	2 (8.7%)	3 (7.0%)	1.00
Low-dose steroids (n, %)	0	9 (39.1%)	21 (48.8%)	0.450
Nintedanib (n, %)	0	1 (4.3%)	1 (2.3%)	1.00

*ACA* anti-centromere antibody, *ACLA* anti-cardiolipin antibody, *ACPA* anti-citrullinated peptide antibody, *AZA* azathioprine, *B2GP1* anti-beta2glycoprotein 1 antibody, *CCBs* calcium channel blockers, *CF* cryofibrinogen/cryofibrinogenemia, *CG* cryoglobulins/cryoglobulinemia, *dsSSc* diffuse cutaneous subset, *DUs* digital ulcers, *ePASP* estimated pulmonary arterial pressure, *ERAs* endothelin receptor antagonists, *HBV* hepatitis B virus, *HCQ* hydroxychloroquine, *HCV* hepatitis C virus, *ILD* interstitial lung disease, *LAC* lupus anticoagulant, *LEF* leflunomide, *MMF*: mycophenolate mofetile *MTX*, metothrexate, *PAH* pulmonary arterial hypertension, *PDE5i* phosphodiesterase 5 inhibitor, *RF* rheumatoid factor, *RNAP3* anti-RNA polymerase 3 antibody, *RP* Raynaud’s Phenomenon, *RTX* rituximab, anti-CD20 antibody, *Scl70* anti-topoisomerase I antibody, *SRC* scleroderma renal crisis

### Subset analysis in patients who did not receive ERA-based therapy, comparison of demographic and clinical characteristics between CF-positive and CF-negative patients and according to CF cryocrit ≥ 1% and < 1%

Among the 54 patients who were not receiving ERA-based therapy (39 CF + and 15 CF-), 46% of CF-positive patients and 13% of CF-negative patients had ePASP ≥ 30 mmHg; the difference was significant (*p* = 0.025) (Table 4).

**Table 4:** Comparison of demographic and clinical characteristics between CF-positive and CF-negative patients who did not receive ERA-based therapy

	Missing	CF-positive (*n* = 39)	CF-negative (*n* = 15)	*p*
Mean age (years ± SD)	0	63.92 ± 13.53	57.53 ± 15.47	0.141
Mean age at diagnosis (years ± SD)	0	55.54 ± 13.59	48.73 ± 13.25	0.103
Mean disease duration (years ± SD)	0	8.72 ± 7.49	9.36 ± 8.18	0.784
Female sex (*n*, %)	0	34 (87.2%)	15 (100.0%)	0.306
Diffuse cutaneous SSc (*n*, %)	0	3 (20.0%)	0 (0.0%)	0.552
CF cryocrit ≥ 1% (*n*, %)	0	12 (30.8%)	-	-
CG positive (*n*, %)	0	5 (12.8%)	0 (0.0%)	0.306
CG cryocrit ≥ 1% (*n*, %)	0	1 (20.0%)	0 (0.0%)	-
RF positive (cut off 15 IU/mL) (*n*, %)	1	8 (21.1%)	2 (13.3%)	0.706
Scl-70 positive (*n*, %)	0	14 (35.9%)	2 (20.0%)	0.338
ACA positive (*n*, %)	0	15 (38.5%)	7 (46.7%)	0.583
Anti-RNAP3 positive (*n*, %)	0	0 (0.0%)	0 (0.0%)	-
Anti-fibrillarin positive (*n*, %)	0	2 (5.2%)	0 (0.0%)	1.00
Anti-U1RNP positive (*n*, %)	0	2 (5.2%)	0 (0.0%)	1.00
Anti-Th/To positive (*n*, %)	0	1 (2.6%)	1 (6.7%)	0.482
Anti-Ku positive (*n*, %)	0	2 (5.1%)	0 (0.0%)	1.00
Anti-MDA5 positive (*n*, %)	0	1 (2.6%)	0 (0.0%)	1.00
Anti-PmScl positive (*n*, %)	0	1 (1.4%)	0 (0.0%)	1.00
AMA positive (*n*, %)	0	3 (7.7%)	2 (13.3%)	0.610
Anti-Ro/SSA positive (*n*, %)	0	2 (5.1%)	2 (13.3%)	0.306
Anti-La/SSB positive (*n*, %)	0	0 (0.0%)	0 (0.0%)	-
ACPA positive (*n*, %)	6	2 (5.9%)	1 (7.1%)	1.00
aPL antibody positive (*n*, %)	0	5 (12.8%)	2 (13.3%)	1.00
ACLA IgM positive (*n*, %)	0	4 (10.3%)	1 (6.7%)	1.00
ACLA IgG positive (*n*, %)	0	0 (0.0%)	1 (6.7%)	0.278
B2GP1 IgM positive (*n*, %)	0	2 (5.1%)	0 (0.0%)	1.00
B2GP1 IgG positive (*n*, %)	0	0 (0.0%)	0 (0.0%)	-
LAC positive (*n*, %)	9	1 (3.0%)	0 (0.0%)	1.00
HCV positive (*n*, %)	23	0 (0.0%)	0 (0.0%)	-
HBV positive (*n*, %)	23	1 (4.8%)	0 (0.0%)	1.00
Presence of ILD (*n*, %)	0	24 (61.5%)	6 (40.0%)	0.154
Presence of ePASP > 30 mmHg (*n*, %)	0	18 (46.2%)	2 (13.3%)	**0.025**
Presence of PAH (*n*, %)	51	2 (100.0%)	0 (0.0%)	0.333
Raynaud’s phenomenon (*n*, %)	0	39 (100.0%)	15 (100.0%)	-
DUs (present/past) (*n*, %)	0	13 (33.3%)	1 (6.7%)	*0.080*
History of amputations (*n*, %)	0	4 (10.3%)	0 (0.0%)	0.567
History of scleroderma renal crisis (*n*, %)	0	1 (2.6%)	0 (0.0%)	1.00
History of calcinosis (*n*, %)	0	6 (15.4%)	1 (6.7%)	0.659
History of arthritis (*n*, %)	0	7 (17.9%)	1 (6.7%)	0.419
Esophageal involvement (*n*, %)	0	26 (66.7%)	9 (60.0%)	0.646
Telangectasias (*n*, %)	0	20 (51.3%)	6 (40.0%)	0.457
History of neoplasm (*n*, %)	0	8 (20.5%)	2 (13.3%)	0.708
Presence of overlap syndrome (*n*, %)	0	10 (25.6%)	5 (33.3%)	0.736
Death (*n*, %)	0	4 (10.4%)	0 (0.0%)	0.567
Prostanoids (*n*, %)	0	34 (87.2%)	12 (80.0%)	0.671
Iloprost (*n*, %)	0	32 (82.1%)	12 (80.0%)	1.00
Alprostadil (*n*, %)	0	2 (5.1%)	0 (0.0%)	1.00
Calcium channel blockers (*n*, %)	0	33 (84.6%)	11 (73.3%)	0.438
Nifedipine (*n*, %)	0	23 (59.0%)	6 (40.0%)	0.210
Amlodipine (*n*, %)	0	8 (20.5%)	4 (26.7%)	0.719
Diltiazem (*n*, %)	0	0 (0.0%)	0 (0.0%)	-
Felodipine (*n*, %)	0	2 (5.1%)	1 (6.7%)	1.00
Antiplatelets (*n*, %)	0	16 (41.0%)	3 (20.0%)	0.147
Low-dose acetylsalicylate (*n*, %)	0	15 (38.5%)	2 (13.3%)	0.106
Ticagrelor (*n*, %)	0	0 (0.0%)	0 (0.0%)	-
Clopidogrel (*n*, %)	0	1 (2.5%)	1 (6.7%)	0.482
Anticoagulant (*n*, %)	0	2 (5.1%)	0 (0.0%)	1.00
Dabigatran (*n*, %)	0	1 (2.5%)	0 (0.0%)	1.00
Apixaban (*n*, %)	0	1 (2.5%)	0 (0.0%)	1.00
Edoxaban (*n*, %)	0	0 (0.0%)	0 (0.0%)	-
Sildenafil (*n*, %)	0	2 (5.1%)	0 (0.0%)	1.00
Immunosuppressants (*n*, %)	0	16 (41.0%)	3 (20.0%)	0.147
MMF (*n*, %)	0	9 (23.1%)	2 (13.3%)	0.708
HCQ (*n*, %)	0	6 (15.4%)	2 (13.3%)	1.00
MTX (*n*, %)	0	1 (2.5%)	1 (6.7%)	0.482
AZA (*n*, %)	0	0 (0.0%)	0 (0.0%)	-
LEF (*n*, %)	0	1 (2.5%)	0 (0.0%)	1.00
RTX (*n*, %)	0	2 (5.1%)	0 (0.0%)	1.00
Low-dose steroids (*n*, %)	0	19 (48.7%)	1 (6.7%)	**0.004**
Nintedanib (*n*, %)	0	1 (2.5%)	0 (0.0%)	1.00

*ACA* anti-centromere antibody, *ACLA* anti-cardiolipin antibody, *ACPA* anti-citrullinated peptide antibody, *AZA* azathioprine, *B2GP1* anti-beta2glycoprotein 1 antibody, *CCBs* calcium channel blockers, *CF* cryofibrinogen/cryofibrinogenemia, *CG* cryoglobulins/cryoglobulinemia, *dsSSc* diffuse cutaneous subset, *DUs* digital ulcers, *ePASP* estimated pulmonary arterial pressure, *ERAs* endothelin receptor antagonists, *HBV* hepatitis B virus, *HCQ* hydroxychloroquine, *HCV* hepatitis C virus, *ILD* interstitial lung disease, *LAC* lupus anticoagulant, *LEF* leflunomide, *MMF* mycophenolate mofetile, *MTX* metothrexate, *PAH* pulmonary arterial hypertension, *PDE5i* phosphodiesterase 5 inhibitor, *RF* rheumatoid factor, *RNAP3* anti-RNA polymerase 3 antibody, *RP* Raynaud’s Phenomenon, *RTX* rituximab, anti-CD20 antibody, *Scl70* anti-topoisomerase I antibody, *SRC* scleroderma renal crisis

No associations were detected between a high cryocrit and any of the investigated characteristics in ERA-negative patients (Table A, supplementary material).

### Relative risk of death and digital amputation according to CF cryocrit

Among 100 patients, 69.0% were CF positive and 31.0% were CF negative; among CF positive patients, 43.0% had a CF cryocrit < 1%, 19.0% had a CF cryocrit of 1%, 1.0% had a CF cryocrit of 2%, 2.0% had a CF cryocrit of 3%, 2.0% had a CF cryocrit of 4%, and 2.0% had a CF cryocrit of 5%.

The prevalence of digital amputation and death were respectively 12.9% and 3.2% for CF negative, 11.6% and 7.0% for CF cryocrit < 1%, 21.1% and 5.3% for CF cryocrit of 1%, 0.0% and 0.0% for CF cryocrit of 2%, 50.0% and 0.0% for CF cryocrit of 3%, 50.0% and 50.0% for CF cryocrit of 4%, and 50.0% and 50.0% for CF cryocrit of 5%.

Among CF positive patients, 8.7% had a cryocrit higher than or equal to 3% and 91.3% had a cryocrit lower than 3%.

**Fig. 1:**
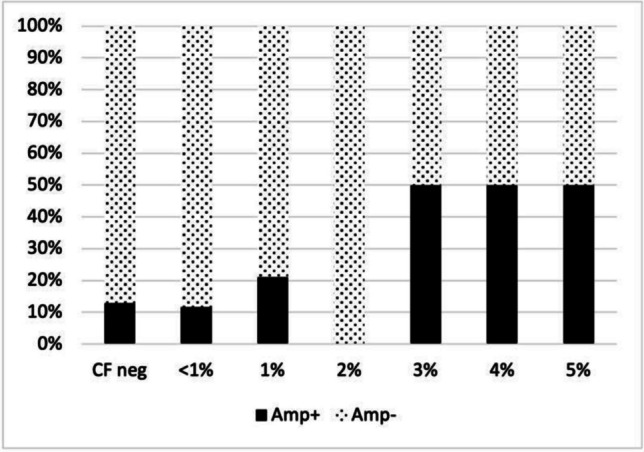
Digital amputation rates as a function of the CF cryocrit. Abbreviations: Amp ± , patients who did/did not undergo digital amputation; CF neg, negative for cryofibrinogen

**Fig. 2:**
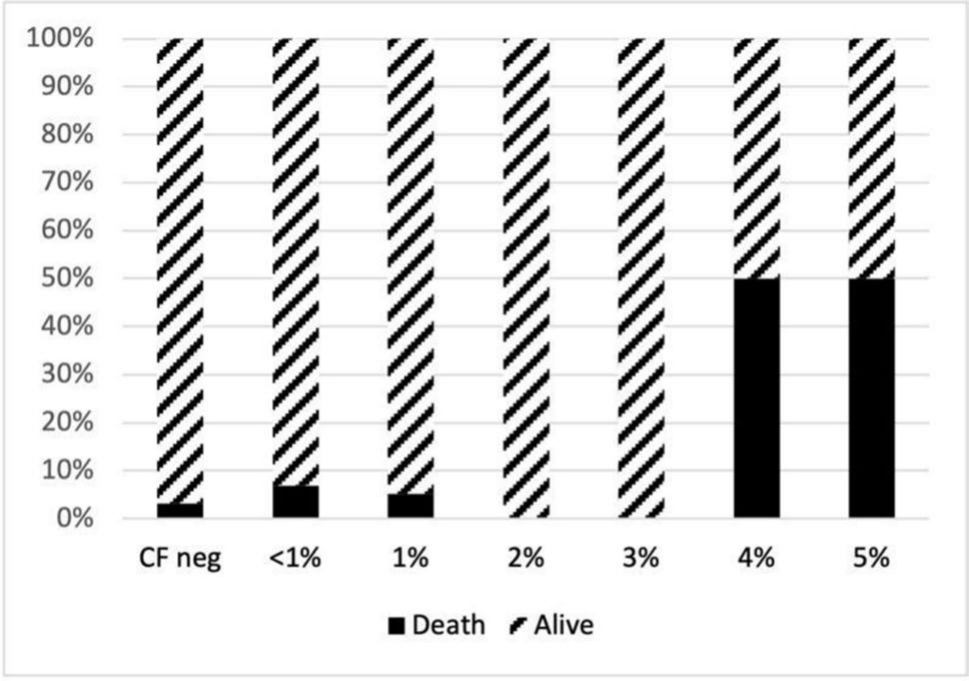
Death rates as a function of the CF cryocrit. Abbreviations: CF neg: negative for cryofibrinogen

The prevalence of digital amputation and death were respectively 50.0% and 33.3% of CF cryocrit ≥ 3%, and 14.5% and 6.5% for CF cryocrit < 3%, with corresponding relative risks of 3.44 (95% CI 1.26–9.39, *p* = 0.016) and 5.17 (95% CI 1.18–22.6, *p* = 0.029) for a cryocrit ≥ 3% (Fig. 1 and Fig. 2).

## Discussion

The current study comprehends the largest SSc cohort in the literature where the CF was evaluated.

Our data confirm the high prevalence of cryofibrinogenemia in SSc patients previously reported in our preliminary study [11] and in literature [19] and confirm the low presence of CGs observed in a previous study [23].

De Almeida et al. retrospectively evaluated the presence of CF in a group of 75 patients affected by SSc and followed up from 2005 to 2018 at Toulouse University Hospital and Joseph Ducing Hospital [19]. A total of 46.6% of patients were positive for CF, lower than the value reported in the present study (69%). This difference may be due to differences in the methods used to select the patients; in our study, patients were screened specifically for cryoproteins regardless clinical aspects, whereas in the French study above, a retrospective analysis of data collected over a long period (75 patients in 13 years) was performed. Moreover, the authors did not specify the reasons – clinical or not – that induced them to test or not cryoproteins, given the small annual case history reported, leading to a possible selection bias.

According to our findings, the presence of CF – either alone or coexisting with CGs – was not associated with a specific clinical phenotype, however, we did find that RNAP3-positive patients were less likely to have cryofibrinogenemia than RNAP3-negative patients. It is known that RNAP3 antibodies are associated to renal involvement in SSc [24]; the only 3 RNAP3 positive patients of our sample present a renal involvement (2 with SRC and 1 with microhematuria and dysmorphic erythrocytes), but since the association with CF is negative, we can assume that the renal damage is not induced by CF presence.

This is the first study where the relationship between vasoactive-based regimens in SSc and CF positivity were investigated. Significant associations between CF positivity and nifedipine use (positive association, *p* = 0.040) and ERAs + PDE5i regimen (negative association, *p* = 0.031), were uncovered. Moreover, from the subset analysis of CF positive patients regarding CF cryocrit emerged an association with the administration of Macitentan and the presence of a higher cryocrit (positive association, *p* = 0.045). These associations could suggest that CF positive patients represent a subset of patients with greater microangiopathic damages and more severe microvascular involvement, requiring the need for a more aggressive vasoactive treatment. Regrettably, our cohort included a limited number of patients who underwent right heart catheterization, therefore this association could not be confirmed, being PAH one of the SSc specific clinical feature related to severe microvascular involvement. Furthermore, a previous case report described the successful management of CF-related acral ulcers through the administration of bosentan in patients affected by non-sclerodermic connective tissue disease [25]. Therefore, it is plausible that the administration of vasoactive drugs for treating microvascular manifestations in SSc (e.g., PAH and DUs) could influence CF or the mechanisms underlying the related microvascular damage.

No other associations between clinical, immunological or immunosuppressant therapies were found by the comparison between neither CF positivity nor a cryocrit equal to or greater than 1% in the entire cohort, in particular no association between ePASP greater than or equal to 30 mmHg. Excluding from the analysis patients treated with ERAs we found that 46.2% of the CF-positive patients and 13.3% of the CF-negative patients had an ePASP greater than or equal to 30 mmHg (*p* = 0.025). These results can suggest that PAH is more common in patients CF positive ERA untreated, since ePASP is an indirect marker of PAH. Moreover, both EULAR [26] and European Society of Cardiology/European Respiratory Society (ESC/ERS) guidelines [27] for PAH recommend the use of ERAs and PDE5i for the management of PAH.

Even if no association emerged between CF positivity and digital amputation and/or death, by the subset analysis according to CF cryocrit, an association with a cryocrit higher than or equal to 3% emerged for both outcomes, and RRs respectively of 3.44 and 5.17 were calculated.

For the first time the association of CF with death and digital amputation in a SSc cohort is demonstrated, as was previously stated in literature but in general population [1–7].

This study has several strengths. First, this is the largest cohort of SSc patients in literature, in which CF is determined; moreover, patients were selected from a third-level SSc European Reference Network (ERN Connect) center.

However, this study also has several limitations, such as its retrospective nature and the lack of right heart catheterization data for a considerable number of patients, making establishing a definite association between CF positivity and PAH not possible.

## Conclusions

Isolated cryofibrinogenemia is a frequent phenomenon observed in SSc patients. CF is associated with a higher administration of vasoactive drugs, probably identifying a SSc clinical phenotype with a more severe microvascular involvement.

Moreover, a higher cryocrit is associated with an increased risk of death and digital amputation, implying a greater systemic involvement.

Our data highlight the need to test CF in all SSc patients, as it could represent an opportunity to provide better therapeutic approaches by anticipating ERA administration in an earlier phase, thereby preventing the manifestation of severe microvascular involvement. Further studies are needed to better investigate the association between the outcomes of SSc patients and ERA-based treatment in the context of cryofibrinogenemia.

Our results underscore the need to evaluate the presence of cryofibrinogen at disease onset, as a possible predictor of specific disease manifestations in a future prospective cohort study. Furthermore, it would be necessary to assess CF in all incident patients with PAH, to determine the possible association with PAH in SSc; if our hypothesis would be confirmed, we could also suggest a screening for CF in all incident patients with non-sclerodermic pulmonary hypertension.

## Supplementary Information

Below is the link to the electronic supplementary material.

Supplementary file1 (DOCX 24.3 KB)

## Declarations

### Open data sharing

The data sets generated during and/or analyzed during the current study are available from the corresponding author upon reasonable request.
