# Supplementary material for: Secondary cryofibrinogenemia is related to more severe microangiopathic involvement in systemic sclerosis: results from a retrospective observational study
Source: Clin Rheumatol. 2025 Jan 20;44(3):1173–85. doi: 10.1007/s10067-025-07324-z (PMC11865222; doi:10.1007/s10067-025-07324-z)
Supplement: Supplementary file 1 — Supplementary file1 (DOCX 24.3 KB) [file 10067_2025_7324_MOESM1_ESM.docx]

**SUPPLEMENTARY MATERIALS**

**Table A: Comparisons between non–ERA-receiving patients with a CF cryocrit ≥1% and those with a CF cryocrit <1%**

|  | Missing | CF cryocrit ≥ 1%  (n = 12) | CF cryocrit < 1%  (n = 27) | p |
| --- | --- | --- | --- | --- |
| Mean age (years±SD) | 0 | 66.67±12.95 | 62.70±13.83 | 0.406 |
| Mean age at diagnosis (years±SD) | 0 | 56.92±13.98 | 54.93±13.64 | 0.679 |
| Mean disease duration (years±SD) | 0 | 10.42±7.71 | 7.97±7.41 | 0.351 |
| Female sex (n, %) | 0 | 10 (83.3%) | 24 (88.9%) | 0.634 |
| Diffuse cutaneous SSc (n, %) | 0 | 1 (8.33%) | 2 (7.4%) | 1.00 |
| CG positive (n, %) | 0 | 2 (16.7%) | 3 (11.1%) | 0.634 |
| CG cryocrit ≥1% (n, %) | 0 | 1 (50.0%) | 0 (0.0%) | 0.317 |
| RF positive (cut off 15 IU/mL) (n, %) | 0 | 2 (18.2%) | 6 (22.2%) | 1.00 |
| Scl-70 positive (n, %) | 0 | 6 (50.0%) | 8 (29.6%) | 0.287 |
| ACA positive (n, %) | 0 | 6 (50.0%) | 9 (29.6%) | 0.478 |
| Anti-RNAP3 positive (n, %) | 0 | 0 (0.0%) | 0 (0.0%) | - |
| Anti-fibrillarin positive (n, %) | 0 | 0 (0.0%) | 2 (7.4%) | 1.00 |
| Anti-U1RNP positive (n, %) | 0 | 1 (8.3%) | 1 (3.7%) | 0.526 |
| Anti-Th/To positive (n, %) | 0 | 0 (0.0%) | 1 (3.7%) | 1.00 |
| Anti-Ku positive (n, %) | 0 | 1 (8.3%) | 1 (3.7%) | 0.526 |
| Anti-MDA5 positive (n, %) | 0 | 0 (0.0%) | 1 (3.7%) | 1.00 |
| Anti-PmScl positive (n, %) | 0 | 0 (0.0%) | 1 (3.7%) | 1.00 |
| AMA positive (n, %) | 0 | 1 (8.3%) | 2 (7.4%) | 1.00 |
| Anti-Ro/SSA positive (n, %) | 0 | 1 (8.3%) | 1 (3.7%) | 0.526 |
| Anti-La/SSB positive (n, %) | 0 | 0 (0.0%) | 0 (0.0%) | - |
| ACPA positive (n, %) | 5 | 1 (10.0%%) | 1 (4.2%) | 0.508 |
| aPL antibody positive (n, %) | 0 | 3 (25.0%) | 2 (7.4%) | 0.159 |
| ACLA IgM positive (n, %) | 0 | 2 (16.7%) | 2 (7.4%) | 0.573 |
| ACLA IgG positive (n, %) | 0 | 0 (0.0%) | 0 (0.0%) | - |
| B2GP1 IgM positive (n, %) | 0 | 1 (8.3%) | 1 (3.7%) | 0.526 |
| B2GP1 IgG positive (n, %) | 0 | 0 (0.0%) | 0 (0.0%) | - |
| LAC positive (n, %) | 6 | 0 (0.0%) | 1 (4.5%) | 1.00 |
| HCV positive (n, %) | 17 | 0 (0.0%) | 0 (0.0%) | - |
| HBV positive (n, %) | 18 | 0 (0.0%) | 1 (6.7%) | 1.00 |
| Presence of ILD (n, %) | 0 | 7 (58.3%) | 17 (63.0%) | 1.00 |
| Presence of ePASP > 30 mmHg (n, %) | 0 | 7 (58.3%) | 11 (40.7%) | 0.309 |
| Presence of ePASP > 35 mmHg (n, %) | 0 | 3 (25.0%) | 6 (22.2%) | 1.00 |
| Presence of PAH (n, %) | 37 | 0 (0.0%) | 2 (100.0%) | - |
| Raynaud’s phenomenon (n, %) | 0 | 12 (100.0%) | 27 (100.0%) | - |
| DUs (present/past) (n, %) | 0 | 5 (41.7%) | 8 (29.6%) | 0.486 |
| History of amputations (n, %) | 0 | 1 (8.3%) | 3 (11.1%) | 1.00 |
| History of scleroderma renal crisis (n, %) | 0 | 1 (8.3%) | 0 (0.0%) | 0.308 |
| History of calcinosis (n, %) | 0 | 1 (8.3%) | 5 (18.5%) | 0.645 |
| History of arthritis (n, %) | 0 | 3 (25.0%) | 4 (14.8%) | 0.654 |
| Esophageal involvement (n, %) | 0 | 7 (58.3%) | 19 (70.4%) | 0.466 |
| Telangectasias (n, %) | 0 | 8 (66.7%) | 12 (44.4) | 0.200 |
| History of neoplasm (n, %) | 0 | 2 (16.7%) | 6 (22.2%) | 1.00 |
| Presence of overlap syndrome (n, %) | 0 | 3 (25.0%) | 7 (25.9%) | 1.00 |
| Death (n, %) | 0 | 1 (8.3%) | 3 (11.1%) | 1.00 |
| Prostanoids (n, %) | 0 | 10 (83.3%) | 24 (88.9%) | 0.634 |
| Iloprost (n, %) | 0 | 10 (83.3%) | 22 (81.5%) | 1.00 |
| Alprostadil (n, %) | 0 | 0 (0.0%) | 2 (7.4%) | 1.00 |
| Calcium channel blockers (n, %) | 0 | 11 (91.7%) | 22 (81.5%) | 0.645 |
| Nifedipine (n, %) | 0 | 5 (41.7) | 18 (66.7%) | 0.174 |
| Amlodipine (n, %) | 0 | 5 (41.7%) | 3 (11.1%) | *0.079* |
| Diltiazem (n, %) | 0 | 0 (0.0%) | 0 (0.0%) | - |
| Felodipine (n, %) | 0 | 1 (8.3%) | 1 (3.7%) | 0.526 |
| Antiplatelets (n, %) | 0 | 7 (58.3%) | 9 (33.3%) | 0.174 |
| Low-dose acetylsalicylate (n, %) | 0 | 7 (58.3%) | 8 (29.6%) | 0.153 |
| Ticagrelor (n, %) | 0 | 0 (0.0%) | 0 (0.0%) | - |
| Clopidogrel (n, %) | 0 | 0 (0.0%) | 1 (3.7%) | 1.00 |
| Anticoagulant (n, %) | 0 | 1 (8.3%) | 1 (3.7%) | 0.526 |
| Dabigatran (n, %) | 0 | 1 (8.3%) | 0 (0.0%) | 0.308 |
| Apixaban (n, %) | 0 | 0 (0.0%) | 1 (3.7%) | 1.00 |
| Edoxaban (n, %) | 0 | 0 (0.0%) | 0 (0.0%) | - |
| Sildenafil (n, %) | 0 | 0 (0.0%) | 2 (7.4%) | 1.00 |
| Immunosuppressants (n, %) | 0 | 5 (41.7%) | 11 (40.7%) | 1.00 |
| MMF (n, %) | 0 | 2 (16.7%) | 7 (25.9%) | 0.693 |
| HCQ (n, %) | 0 | 3 (25.0%) | 3 (11.1%) | 0.348 |
| MTX (n, %) | 0 | 0 (0.0%) | 1 (3.7%) | 1.00 |
| AZA (n, %) | 0 | 0 (0.0%) | 0 (0.0%) | - |
| LEF (n, %) | 0 | 1 (8.3%) | 0 (0.0%) | 0.308 |
| RTX (n, %) | 0 | 0 (0.0%) | 2 (7.4%) | 1.00 |
| Low-dose steroids (n, %) | 0 | 5 (41.7%) | 14 (51.9%) | 0.731 |
| Nintedanib (n, %) | 0 | 0 (0.0%) | 1 (3.7%) | 1.00 |

Abbreviations: ACA: anti-centromere antibody; ACLA: anti-cardiolipin antibody; ACPA: anti-citrullinated peptide antibody; AZA: azathioprine; B2GP1: anti-beta2glycoprotein 1 antibody; CCBs: calcium channel blockers; CF: cryofibrinogen/cryofibrinogenemia; CG: cryoglobulins/cryoglobulinemia; dsSSc: diffuse cutaneous subset; DUs: digital ulcers; ePASP: estimated pulmonary arterial pressure; ERAs: endothelin receptor antagonists; F: female sex; HBV: hepatitis B virus; HCQ: hydroxychloroquine; HCV: hepatitis C virus; ILD: interstitial lung disease; LAC: lupus anticoagulant; LEF: leflunomide; MMF: mycophenolate mofetile; MTX: metothrexate; PAH: pulmonary arterial hypertension; PDE5i: phosphodiesterase 5 inhibitor: RF: rheumatoid factor; RNAP3: anti-RNA polymerase 3 antibody; RP: Raynaud’s Phenomenon; RTX: rituximab, anti-CD20 antibody; Scl70: anti-topoisomerase I antibody; SRC: scleroderma renal crisis.
